# Glandular trichomes: new focus on horticultural crops

**DOI:** 10.1038/s41438-021-00592-1

**Published:** 2021-07-01

**Authors:** Zhongxuan Feng, Ezra S. Bartholomew, Ziyu Liu, Yuanyuan Cui, Yuming Dong, Sen Li, Haoying Wu, Huazhong Ren, Xingwang Liu

**Affiliations:** 1 Engineering Research Center of the Ministry of Education for Horticultural Crops Breeding and Propagation, College of Horticulture, China Agricultural University, 100193 Beijing, P. R. China; 2 Beijing Key Laboratory of Growth and Developmental Regulation for Protected Vegetable Crops, College of Horticulture, China Agricultural University, 100193 Beijing, P. R. China; 3 Library of China Agricultural University, China Agricultural University, 100193 Beijing, P. R. China; 4 State Key Laboratory of Vegetable Germplasm Innovation, Tianjin, China

**Keywords:** Plant development, Plant molecular biology

## Abstract

Plant glandular trichomes (GTs) are epidermal outgrowths with the capacity to biosynthesize and secrete specialized metabolites, that are of great scientific and practical significance. Our understanding of the developmental process of GTs is limited, and no single plant species serves as a unique model. Here, we review the genetic mechanisms of GT initiation and development and provide a summary of the biosynthetic pathways of GT-specialized metabolites in nonmodel plant species, especially horticultural crops. We discuss the morphology and classification of GT types. Moreover, we highlight technological advancements in methods employed for investigating GTs. Understanding the molecular basis of GT development and specialized metabolites not only offers useful avenues for research in plant breeding that will lead to the improved production of desirable metabolites, but also provides insights for plant epidermal development research.

## Introduction

Trichomes, the specialized structures that cover most aerial plant tissues, are classified as glandular or nonglandular based on their morphology and secretion ability^1^. Glandular trichomes (GTs) are described as biofactories with the unique capacity to biosynthesize specialized metabolites, which are critical for the capacity of plants to adapt to their environment and to overcome biotic and abiotic stresses^2,3^. Moreover, several metabolites produced by medicinal plant GTs are exploited by pharmaceutical industries that benefit from their psychoactive, antiparasitic, antitumor, antimicrobial, antiviral, and antithrombotic properties^4^.

**Fig. 1:**
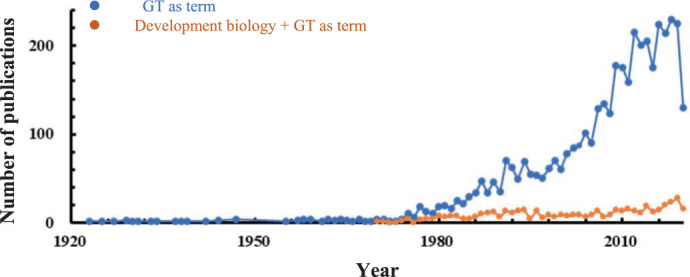
Numbers of glandular trichome-related publications from 1900 to 2020. (Based on the Web of Science database using the search word “glandular trichome”)

GT engineering is a plant breeding strategy that requires a detailed understanding of the genetic network controlling GT development^3,5^. Literature searches using the keyword “glandular trichome” generated approximately 4200 published articles on the Web of Science database between 1900 and 2020. Over 70% of these articles focused on the biochemistry, anatomy, and morphology of GTs, whereas 11% were related to their developmental biology (Fig. 1). Our understanding of the genetic mechanisms of GT development is still in its infancy. However, research progress has been sharply and steadily increasing over the past 15 years. This period coincides with the discovery of the first gene involved in the biosynthesis of metabolites from mint and the increased availability of genetic resources from plants, especially horticultural crops, such as *Artemisia annua* L. (sweet wormwood) and *Cucumis sativus* L. (cucumber)^6,7^.

Research on unicellular nonglandular trichomes in the model plant *Arabidopsis thaliana* has been abundant. As no GTs have been found in *A. thaliana*, research on GT development has been conducted in other plant species, including vegetable crops, floral crops, medical plants, aromatic crops and so on. However, no single plant species serves as a unique model for multicellular GTs. From careful analysis of recent literature, five plant species emerged as key working materials: tomato (*Solanum lycopersicum*), cucumber (*C. sativus*), sweet wormwood (*A. annua*), tobacco (*Nicotiana tabacum*), and cotton (*Gossypium hirsutum*). Recent studies on these plant species that have led to significant advances in our understanding of GT morphology and developmental biology are summarized in this review. Furthermore, we discuss current advances and innovations applied in the study of GT development and provide future perspectives on the application of current knowledge to enhance breeding efforts.

### Morphology and classification of glandular trichomes

**Fig. 2:**
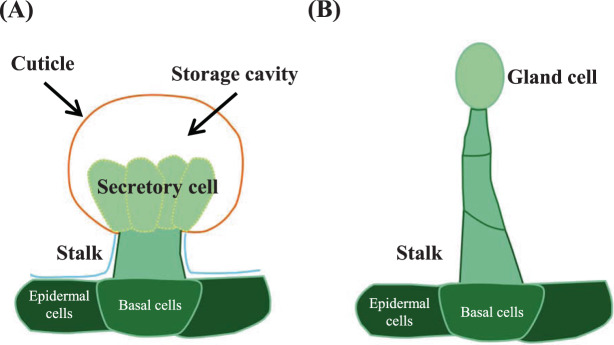
Types of glandular trichomes. Schematic diagrams showing a peltate trichome (**A**) and capitate trichome (**B**)

GTs are extremely diverse in terms of shape, cell number, and type of secreted metabolites and may be the result of different evolutionary events^3^. The morphology of GTs has been described in many plant species, and there are excellent reviews that highlight their diversity^3^. Briefly, GTs are typically multicellular, consisting of a head/gland that secretes specialized metabolites, a stalk that supports the head, and a differentiated base that connects the stalk to surrounding epidermal cells. There are two main types of GTs, namely, peltate and capitate, with differences in morphology and structure^8^. Peltate trichomes are short with unicellular or bicellular stalks and a large multicellular secretory head containing several secretory cells, whereas capitate trichomes typically consist of a multicellular stalk of variable cell number and length with a smaller unicellular head^8^ (Fig. 2). Here, we briefly describe the morphology and classification of GTs in relevant plant species.

Cultivated tomato and its wild relatives contain four types (I, IV, VI, and VII) of GTs^9^. Types I and IV are capitate trichomes, while types VI and VII are peltate trichomes. Type I trichomes are typically longer than type IV trichomes and are present in several tomato species, while type IV trichomes are commonly found in wild species such as *S. pennellii* and *S. habrochaites*^10^. Type VI GTs represent the most abundant trichome type on the leaves and stems of tomato plants and contain four-celled secretory heads with an intercellular storage cavity that can accumulate metabolites. The development and shape of the type VI glandular trichome head differ between cultivated tomato and the wild species *S. habrochaites*^11^.

*A. annua* contains two types of trichomes, known as glandular secreting trichomes (GSTs) and T-shaped nonglandular trichomes (TNGs)^12^. *A. annua* GST (AaGST) has received much attention due to its capacity to biosynthesize artemisinin, an effective medicine used in malaria treatment^13–16^. The AaGSTs in which artemisinin biosynthesis and accumulation occur in a 10-celled biseriate structure are composed of two apical cells, four subapical cells, two stalk cells and two basal cells^16,17^.

Cucumber fruit is covered by trichomes that combine with tubercles to form the warty fruit trait, which is an important quality trait in cucumber production^18,19^. Xue et al. examined fruit trichomes of 200 cucumber varieties and classified them into eight distinct types (I–VIII), of which types I and VI are GTs^18^. Type I trichomes are peltate with a short stalk composed of 3–4 cells and a four-celled or five-celled head^18^ and are the most widespread and studied GTs found on almost all cucumber species. Although most research on cucumber trichomes has focused on the fruit, type I and VI trichomes can also be found on leaves, stems, flowers, and tendrils^18,19^.

Tobacco contains two types of capitate trichomes: long trichomes with a multicellular stalk possessing unicellular or multicellular heads and short trichomes with a unicellular stalk and a multicellular head^20^. Long trichomes synthesize and secrete several exudates and metabolites^21^, including terpenoids and calcium oxalate crystals^22^, whereas short trichomes are hydathodes that secrete aqueous droplets under conditions of high atmospheric humidity^21^. Nicotine (dissolved in aqueous droplets) and heavy metals, including Cd and Zn, are extruded by short trichomes^23^. Short trichomes are also regarded as specialized biosynthetic structures that produce and secrete defense-related proteins known as *T-phylloplanins* to aerial leaf surfaces^24^.

Unlike other species, cotton species are characterized by the presence of darkly pigmented lysigenous glands, which are also called gossypol glands, black glands, or oil glands; these glands are located in the subepidermal layer of many plant tissues and originate from a cluster of cells in the ground meristem^25–27^. These glands contain high-density gossypol and related terpenoids, which defend the plant from biotic and abiotic stresses and are toxic to monogastric animals^25–27^. The whole gossypol gland is composed of modified nucleus-free secretory cells with a large vacuole and drastic cytoplasmic structural disorganization, that is, bounded by one layer of secretory cells and one to three layers of sheath cells^27^. Although glands on cotton show different structures from other species, in-depth knowledge of their molecular regulation is a good way to help obtain a clear view of plant GT development.

Cannabis (*Cannabis sativa*) and Japanese catnip (*Schizonepeta tenuifolia* Briquetare) also contain trichomes of biotechnological interest. The female cannabis flower contains three types of GTs: sessile, bulbous and stalked. Sessile trichomes are similar to peltate trichomes, while stalked trichomes are similar to capitate trichomes^28,29^. These GT types also differ in their fluorescent properties, the number of secretory cells and terpene metabolite profiles^28^. Japanese catnip, in traditional Asian medicine, contains three distinct GT types, namely, peltate, capitate, and digitiform, with peltate trichomes being the main site for the biosynthesis of essential oil^30^.

### Genetic mechanisms of glandular trichome development

**Fig. 3:**
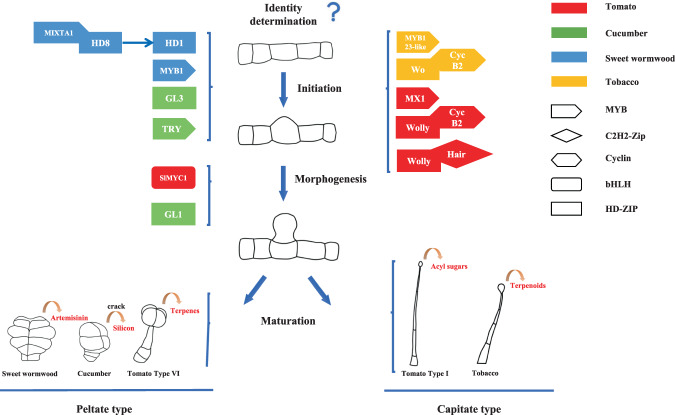
Model of glandular trichome development. Schematic diagrams showing GTs of tomato, cucumber, sweet wormwood and tobacco, as well as their main molecular development regulation pathway

Considerable interest in bioactive compounds produced by GTs, combined with the application of multiomic technologies (genomics, transcriptomics, proteomics, and metabolomics), has greatly accelerated our understanding of gene regulatory networks that function in GT formation^1^. The development of GTs can be roughly divided into four stages: identity determination, initiation, morphogenesis, and maturation. Given their common organization scheme, it is suggested that some GTs share similar developmental events^5^. For example, the initiation of most GTs is regulated by MYB transcription factors. Furthermore, most capitate trichomes, such as tomato type I GTs and tobacco GTs, are typically regulated by the interaction of cyclins and homeodomain-leucine zipper (HD-ZIP) transcription factors (TFs). However, peltate trichomes, such as tomato type VI GTs, are also regulated by bHLH TFs (Fig. 3). Recent studies have characterized several genes involved in GT development in key plant species (Fig. 3 and Table 1), which are summarized in this review.

**Table 1:** Genes involved in the development of GTs

Types	Species	TFs Name	Function	Metabolites production	Binding sites	Interaction Genes	Hormone involved	Reference
AP2/ERF	Sweet wormwood	*TAR1*	GST morphogenesis	Artemisinin				^14^
bHLH	Cotton	*GoPGF/CGF3*	Pigment gland formation	Gossypol	G-box	*TPSs, WRKYs*	JA	^25,61^
		*CGF1*	Pigment gland morphogenesis					^25^
	Tomato	*SlMYC1*	Type VI formation	Terpenoids		*TPSs*		^49^
MYB	Sweet wormwood	*AaMYB1*	GST density	Artemisinin			GA	^31^
		*AaMIXTA1*	GST density	Artemisinin		cuticle biosynthesis genes		^32^
	Cotton	*CGP1*	Pigment gland pigmentation	Gossypol		*GoPGF*		^26^
	Tomato	*SlMX1/SlMIXTA-like*	Glandular trichome density (especially type I)	Terpenoids, carotenoids, and phenylpropanoids	MRE			^50–52^
	Cucumber	*CsTRY*	Trichome dentsity			CsMYB6		^43^
	Tobacco	*NbMYB123-like*	Glandular trichome density					^67^
NAC	Cotton	*CGF2*	Pigment gland density	Gossypol				^25^
HD-ZIP	Tomato	*Wolly*	Type I density	Terpenoids		*SlCycB2*		^44,45^
	Tobacco	*Nbwo*	Glandular trichome formation		L1-like	*NbCycB2,Nbwo*		^66^
	Sweet wormwood	*AaHD1*	GST density	Artemisinin		AaJAZ8	JA	^34^
	Sweet wormwood	*AaHD8*	GST density	Artemisinin	L1-box/HZBS	*AaHD1*, AaMIXTA1, cuticle biosynthesis genes		^33^
	Cucumber	*CsGL3/ Tril*	Trichome initiation					^40–42^
	Cucumber	*CsGL1/Mict/Tbh*	Trichome morphogenesis			CsMYB6, CsGA20ox1		^36–39^
ZFPs	Tomato	*Hair*	Type I formation			*Wolly*		^46^
	Sweet wormwood	*AaSAP1*	GST density	Artemisinin			JA, ABA, GA	^35^
	Tobacco	*NbGIS*	Glandular trichome density				GA	^67^
Aux ⁄ IAA	Tomato	*SlIAA15*	Type I, VI density				auxin	^58^
ARF	Tomato	*SlARF3*	Type I, VI density				auxin	^57^
WD-repeat protein	Cucumber	*CsTTG1*	Trichome density			CsGL1/Mict/Tbh		^43^
Cyclin	Tomato	*SlCycB2*	Glandular trichome density (especially Type I)	Terpenoids				^44,45^
	Tobacco	*NbCycB2*	Glandular trichome formation			Nbwo		^66^
WAVE regulatory complex	Tomato	*SRA1*	Glandular trichome morphogenesis	Flavonoids, sesquiterpenoids				^55^
CHI	Tomato	*CHI1*	Glandular trichome density	Flavonoids				^54^
JA-responsive	Tomato	*JAI-1*	Type I,VI formation				JA	^59^

### Mechanisms in sweet wormwood (*A. annua*)

In *A. annua*, *TRICHOME AND ARTEMISININ REGULATOR 1* (*TAR1*), encoding an AP2 TF, plays a crucial role in regulating the development of GTs and the biosynthesis of artemisinin^14^. MYB TFs, including AaMYB1, are known to positively regulate the development of AaGSTs^31^. AaMIXTA1, encoding an R2R3MYB TF, interacts with AaHD8, an HD-ZIP IV TF, forming a regulatory complex that directly promotes AaHD1 expression and positively regulates the initiation of GTs^32–34^. Furthermore, *AaSAP1* encodes stress-associated protein 1 (SAP1), which positively regulates the development and density of AsGSTs and the production of artemisinin^35^.

### Mechanisms in cucumber (*C. sativus*)

Understanding the molecular genetic basis of fruit spine development is a key aspect of cucumber research^18^. In cucumber, several trichome-related mutants have been reported. Chen et al. characterized the *tiny branched hair* (*tbh*) mutant, which had no noticeable fruit spines but contained tiny branched trichomes with reduced cell numbers and aberrant cell shapes and organization^36^. Li et al. identified *C. sativus Glabrous 1* (*CsGL1*), encoding an HD-Zip I TF, which plays a significant role in cucumber trichome formation. The *csgl1* mutant had no noticeable spines but contained papillae on the leaf epidermis, as observed by scanning electron microscopy (SEM). Furthermore, *CsGL1* indirectly regulates the expression of *CsMYB6* and *CsGA20ox1*^37^. Zhao et al. characterized a *micro-trichome* (*mict*) mutant with microsized stunted spines similar to those of *csgl1*^38,39^. Notably, *TBH*, *MICT*, and *CsGL1* are allelic and mapped to *Csa3M748220*^37,39^. Pan et al. characterized a completely glabrous mutant with a single recessive gene named *C. sativus Glabrous 3* (*CsGL3*), encoding an HD-Zip IV TF^40^. Cui et al. identified the glabrous mutant *NCG157* and postulated its candidate gene to be *Csa6M514870*, which is also the candidate gene for *CsGL3*^41^. Wang et al. demonstrated that *Tril* (allelic to *CsGL3*) had a long segment insertion following the first exon and that the *tril* mutant displayed the same phenotype as *csgl3*^42^. Moreover, *CsGL3*/*Tril* had an epistatic effect on *TBH/CsGL1*/*Mict*^40,42^. Trichome density was shown to be influenced by the expression of *CsTTG1*, which encodes a WD-repeat protein^43^. The genes listed above play a role in both cucumber GTs and nonglandular trichomes; however, little is known about genes directly involved in the development of cucumber GTs.

### Mechanisms in tomato (*S. lycopersicum*)

In tomato, the formation of type I capitate GTs involves the *Wolly* (*Wo*) gene, encoding an HD-ZIP TF, together with *SlCycB2*. The repression of *Wo* has been shown to decrease the number of type I trichomes^44,45^. Additionally, the *Hair (H)* gene, encoding a C2H2 zinc finger protein, interacts with Wo to form an H-Wo dimer protein complex, which acts as an important regulator of type I trichomes^46^. This suggests that H-Wo-CycB2 may form a trimer protein complex that acts as an important regulator of type I trichomes. This differs from the formation of unicellular nonglandular trichomes in *A. thaliana*, which is regulated by an MYB-bHLH-WD trimer complex^46,47^. Recently, the long noncoding RNA (lncRNA) lncRNA000170 was reported to inhibit type I trichome formation. Overexpression of lncRNA000170 caused a decrease in type I trichomes by downregulating several trichome regulators, including *Wo*, *H*, and *SlCycB2*^48^. The formation of type VI peltate trichomes involves *SlMYC1*, encoding a bHLH TF^49^. Overexpression of *SlCycB2* also led to a decrease in type VI GTs^44^. Ectopic expression of *SlMIXTA1*, an R2R3MYB TF, increased the number of GTs, especially type I GTs, in tomato^50–52^. The identification and characterization of several tomato trichome-related mutants have aided the elucidation of the mechanisms of GT formation. The tomato odorless-2 (*od-2*) mutant had abnormal type I trichomes and a low density of type VI trichomes^53^. The *anthocyanin-free* (*af*) mutant, with mutation of *SlCHI*, an isoform of the flavonoid biosynthetic enzyme chalcone isomerase (CHI), exhibited a lower density of type VI trichomes^54^. Kang et al. used a map-based cloning approach to demonstrate that a previously reported *hairless* (*hl*) mutant, controlled by a highly conserved SRA1 (specifically Rac1-associated protein) subunit of the WAVE regulatory complex (WRC), showed a severely bent and shortened trichome phenotype^55,56^. Furthermore, several phytohormone-related genes are involved in the development of GTs, such as *JAI-1*, *SlIAA5*, and *SlARF3*^57–59^, and treatment with exogenous JA was shown to increase the densities of type VI trichomes^60^.

### Mechanisms in cotton (*Gossypium* spp.)

Understanding the molecular genetic basis of gossypol gland formation in cotton could provide additional methods for developing gossypol-free cotton seeds or decrease the density of gossypol glands^61^. Six independent loci, *gl*_*1*_–*gl*_*6*_, that regulate gossypol gland formation have been identified, with the combination of *gl*_*2*_*gl*_*2*_*gl*_*3*_*gl*_*3*_ producing a glandless phenotype^62^. A glandless mutant discovered in Egyptian cotton (*G. barbadense*) was formed due to the expression of a dominant allele at the *Gl*_*2*_ locus, which is epistatic to Gl_3_ and designated GL_2_^e^ ^63,64^. Using the Gl_2_^e^ mutant, Ma et al. characterized *GoPGF* (pigment gland formation) on chromosome A12 as *Gl*_*2*_ and its homeolog on chromosome D12 as *Gl*_*3*_^61^. *GoPGF* encodes a basic helix-loop-helix domain-containing TF that positively regulates gland formation. The insertion of a single nucleotide into the coding sequence (CDS) of *GoPGF* resulted in premature translation termination, leading to the glandless phenotype, while silencing *GoPGF* led to a completely glandless phenotype^61^. The glandless gene Gl_2_^e^ was fine mapped to a 15 kb region, and *GoPGF* was confirmed as the candidate gene^65^. RNA-seq analysis of embryos from near-isogenic gland (*Gl*_*2*_*Gl*_*2*_*Gl*_*3*_*Gl*_*3*_) vs. glandless (*gl*_*2*_*gl*_*2*_*gl*_*3*_*gl*_*3*_) cotton plants identified three *cotton gland formation* (*CGF*) genes that participate in gland formation^25^. The sequences of *CGF1* and *CGF2* in the glandular and glandless cotton plants were identical. However, the A subgenome of *CGF3* (synonymous *GoPGF*) in the glandless cotton had a 5.1 kb transposon insertion, while the D subgenome homolog had two SNPs in the CDS, one SNP in the terminator, and several major differences in the upstream regulatory sequence (~4.2 kb). Silencing of *CGF1* and *CGF3* resulted in a dramatic reduction in gland numbers, while *CGF2* had a mild effect on gland density^25^. In another recent study, Gao et al. performed comparative transcriptome analysis of several glandular and glandless cultivars and further characterized Cotton Gland Pigmentation 1 (*CGP1*), an MYB TF, involved in the regulation of gland pigmentation but not morphogenesis. CGP1 is located in the nucleus and interacts with GoPGF^26^.

### Mechanisms in tobacco (*N. tabacum*)

Recent research in *N. benthamiana* has led to the discovery of a novel reciprocal regulation mechanism that is involved in GT formation^66^. Wu et al. cloned two tobacco genes, namely, *NbCycB2* and *NbWo* (homologs of *SlCycB2* and *SlWo*), and demonstrated that *NbWo* directly regulated the expression of *NbCycB2* by binding to an L1-like box in its promoter region^66^. Wu et al. also suggested that NbCycB2 can inhibit trichome initiation by binding to the LZ domain of NbWo^66^. Furthermore, two C2H2 zinc finger TFs, namely, *NbGIS* and *NbMYB123*-like (homologs of *AtMYB123*), encoding an R2R3 MYB domain putative TF, also participate in the development of tobacco GTs^67^.

### Biosynthesis pathways of glandular trichome specialized metabolites

GTs are sites for the biosynthesis and accumulation of a wide range of plant natural products, such as cannabinoids and terpenes in cannabis^28^, bitter acid in *Humulus lupulus*^68^, tanshinone in *Salvia miltiorrhiza*^69^, and artemisinin in *A. annua*^70^. The cracks of cucumber GTs have also been shown to induce the excretion of silicon^71^. Recent advancements in multiomic technologies and metabolic analysis have shed some light on key molecular pathways regulating the biosynthesis of GT-specialized metabolites.

**Fig. 4:**
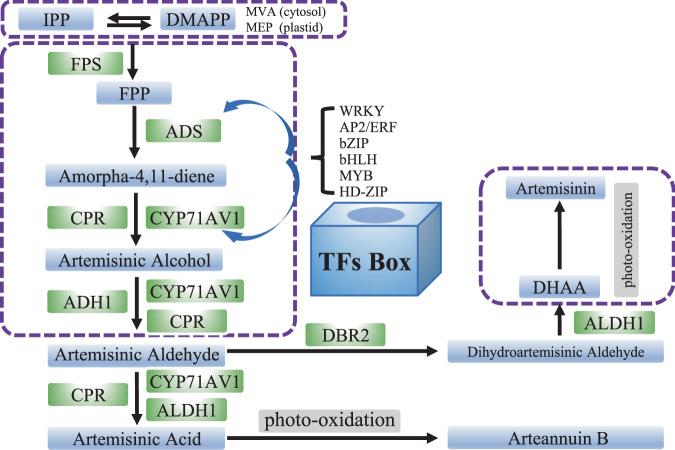
Regulation of artemisinin metabolism in sweet wormwood. TFs involved in the biosynthesis of artemisinin by interaction with the key enzymes in *A. annua*

Due to its importance in the pharmaceutical industry, the mechanism of artemisinin biosynthesis is well described (Fig. 4)^72–75^. Artemisinin originates from isopentenyl diphosphate (IPP) or dimethylallyl diphosphate (DMAPP) via the methylerythritol phosphate (MEP) pathway in the plastid or via the mevalonate (MVA) pathway in the cytosol^70^. The first substrate of the artemisinin biosynthesis pathway is farnesyl diphosphate (FPP), which is synthesized from IPP and DMAPP by farnesyl diphosphate synthase (FPS)^76,77^. The cyclization of FPP to amorpha-4,11-diene by amorpha-4,11-diene synthase (ADS) is the initial step of artemisinin biosynthesis^78,79^. Cytochrome P450 monooxygenase (CYP71AV1), cytochrome P450 oxidoreductase (CPR) and alcohol dehydrogenase (ADH1) then convert ADS to artemisinic alcohol and eventually to artemisinic aldehyde^80–82^. Artemisinic aldehyde Δ11(13)-reductase (DBR) is then involved in the formation of dihydroartemisinic aldehyde (DHAAA)^83^, and aldehyde dehydrogenase 1 (ALDH1) converts DHAAA to dihydroartemisinic acid (DHAA)^84^. DHAA is then converted to artemisinin through photooxidation in the GT subcuticular space. Artemisinic acid is also converted to artemisinin B by photooxidation. Most of these key enzymes are specifically localized in AaGSTs^17,85^. The low yield of artemisinin (0.01–0.1% leaf DW) in *A. annua* severely restricts its supply^86^, and the overexpression of key genes in AaGSTs is an effective strategy for enhancing the artemisinin level. Several TF families are involved in the regulation of artemisinin biosynthesis, including the WRKY (AaWRKY1 and AaGSW1), AP2/ERF (AaORA, AaERF1, AaERF2, and TAR1), bZIP (AabZIP1 and AaHY5), bHLH (AaMYC2 and AabHLH1), MYB (AaMYB1 and AaMIXTA1), HD-ZIP (AaHD1 and AaHD8), and ZFP (AaSAP1) families. Many of these TFs are regulated by phytohormones such as GA, JA, MeJA, and ABA. These TFs regulate artemisinin biosynthesis by interacting with key enzymes, such as DBR2, ADS, and CYP71AV1, or with other TFs^14,31–35,87–95^.

Understanding the genetic basis of gossypol biosynthesis could provide additional methods for the development of gossypol-free cotton seeds^27^. Several enzymes of the gossypol biosynthesis pathway have been characterized, including 3-hydroxy-3-methylglutaryl coenzyme A reductase (HMGR), farnesyl diphosphate synthase (FPS), (+)-δ-cadinene synthase (CDN), (+)-δ-cadinene-8-hydroxylase CYP706B1 and methyltransferase^96^. However, Ma et al. discovered that gossypol biosynthesis and pigment gland formation are uncoupled, as silencing *CYP706B1* does not affect gland formation^61^.

Tomato type VI GTs are the main site of terpenoid, flavonoid and methyl ketone secretion, whereas type I and type IV GTs are involved in acyl sugar biosynthesis^10,55,97^. Overexpression of *SlMX1*/*SlMIXTA*-like increases the expression of genes involved in primary metabolic pathways, such as glycolysis, the pentose phosphate pathway and the shikimate pathway. *SlMX1*/*SlMIXTA*-like is also involved in the biosynthesis of terpenoids and carotenoids^50,52^. Genes that regulate glandular trichome formation, such as *SlMYC1*, *Wolly*, and *SlCycB2*, can also regulate terpenoid biosynthesis and the expression of terpene synthase (TPS) genes^44,45,49^. Mutations in *Od-2*, *SlCH1*, and *SRA1* prevent the accumulation of both flavonoids and terpenoids in type VI glands^53–55^.

### Specialized techniques applied in the study of glandular trichomes

Advanced biotechnologies employed in the study of GTs have assisted researchers in making breakthroughs in understanding the genetics, molecular basis, and functions of GTs and their metabolites. Early research on the separation and purification of GTs was generally ineffective and imprecise. Techniques such as freezing and powdering of plant samples followed by vortexing and mesh filtration were used to collect stalked GTs. In tobacco, direct extraction with tweezers was used to separate trichomes. In tomato, a pulled Pasteur pipette was used to collect type VI glands and their exudate on stems and leaves^49,59^. In peppermint and spearmint, a combination of chemical and physical methods was utilized, involving the application of an isolation buffer to protect the plant materials^98^. Gradually, this method was modified and then widely implemented in several other species^99^. In tomato, GTs were also isolated using glass beads in a chemical isolation buffer, followed by filtration and centrifugation to separate trichome types^10^. This method was adapted to harvest enriched cannabis trichome fractions^28^.

Single-cell data are useful for elucidating cell type-specific processes, cell differentiation and the evolution of cell states^100^. Currently, laser capture microdissection (LCM) is used to isolate and purify single trichomes or secretory cells from paraffin-embedded plant tissue sections. However, LCM is time consuming and inefficient at collecting data from a large number of trichomes. Single-cell RNA sequencing technology is considered an efficient method for identifying cell differentiation states and has been used to study root cell development, vascular cell lineage and stomatal lineage cells^101–104^. Laser microdissection and pressure catapulting (LMPC) is used to isolate single cells from trichomes, and further proteinase K treatment improves RNA yields for downstream analysis^105^. In tomato, fluorescence-activated cell sorting (FACS) based on the autofluorescence of trichomes was used to separate trichrome developmental stages^100^. To observe trichome density and morphology, SEMs and stereoscopes are typically used^11,18^. In *A. annua*, an Olympus fluorescence microscope was used to observe trichome density^35^, while light and fluorescence microscopy analyses were applied in tomato^10^. Furthermore, metabolites stored in GTs can be stained with chemical reagents to observe their secretion process^106^. Metabolites in GTs have been measured using analytical techniques such as GC-MS, HPLC, UPLC, LC-MS, and LC-ESI-MS/MS^14,26,28,33,61^. Currently, internal electrode capillary pressure probe electrospray ionization mass spectrometry (IEC-PPESI-MS) is used for single-cell metabolite profiling of stalk and glandular cells of intact trichomes in tomato, enabling high-spatial-resolution cell sampling, precise postsampling manipulation, and high detection sensitivity^107^.

### Concluding remarks and future perspectives

Specialized metabolites biosynthesized by GTs are considered important reservoirs of high-value bioactive natural products with largely unexploited potential. Despite extensive studies on GT morphology and specialized metabolites, almost nothing is known about the genetics underlying their development. Furthermore, identity determination of GTs is a significant stage, but the developmental signals that initiate the transformation of an epidermal cell to an epidermal hair, and regulate the differentiation of an epidermal hair cell to glandular or nonglandular trichomes are still not clear in most species. Recent advancements in multiomic technologies, genetic resources and specialized techniques have increased our understanding of the genetic mechanisms controlling GT initiation and development in several key plant species. Single-cell metabolite profiling of GTs provides significant results but is difficult to widely apply in diverse plants. In addition, the marker genes related to each developmental stage of GTs are still relatively unknown. Increased knowledge of GT biology and further improvements in these technologies not only will improve our understanding of cell differentiation and the development of plant trichomes, but also could inspire breeding efforts to utilize plants as biofactories that produce desirable metabolites in their GTs.
